# A case of intramural esophageal dissection caused by vomiting during barium esophagography

**DOI:** 10.1002/deo2.70081

**Published:** 2025-02-14

**Authors:** Takuya Ogiso, Shuji Ikegami, Tomohiko Matsuba, Yusuke Takeuchi, Masahiro Takayanagi

**Affiliations:** ^1^ Department of Gastroenterology Chutoen General Medical Center Shizuoka Japan

**Keywords:** dissection cavity, esophageal cancer, esophagography, intramural esophageal dissection, submucosal dissection

## Abstract

Intramural esophageal dissection is a rare disease characterized by a laceration of the submucosal layer in the esophageal wall due to mechanical damage or esophageal pressure, resulting in the separation of the mucosal layer from the muscularis layer. This report presents a case of intramural esophageal dissection induced by vomiting during barium esophagography in a 70‐year‐old man undergoing evaluation for esophageal cancer. Preoperative assessments included endoscopic biopsies and the placement of a marking clip, followed by barium esophagography. During the procedure, the patient experienced vomiting and subsequent neck‐chest pain. Computed tomography revealed a barium‐filled dissection cavity within the esophagus. Esophagogastroduodenoscopy identified submucosal dissection proximal to esophageal cancer, exposing the muscularis layer and the entry site was at the same level as the biopsy site. The patient was treated conservatively with fasting, leading to symptom resolution. Follow‐up endoscopic evaluations confirmed that the entry site remained open but epithelialized, and the esophageal wound had healed.

## INTRODUCTION

The esophagus is easily damaged by various causes including vomiting, and Mallory‐Weiss syndrome (esophageal superficial mucosal lacerations) and Boerhaave syndrome (esophageal transmural perforation) are well‐known examples. Intramural esophageal dissection (IED) is a rare disease characterized by tissue detachment in the submucosal layer.^1^ Although, there are some reports caused by hematoma in the submucosa,^2^, ^3^, ^4^, ^5^ to the best of our knowledge, IED induced by barium esophagography has yet to be clearly described. Herein, we report a case of IED in which the wound process was evaluated over time from early onset by esophagogastroduodenoscopy (EGD).

## CASE REPORT

**FIGURE 1:**
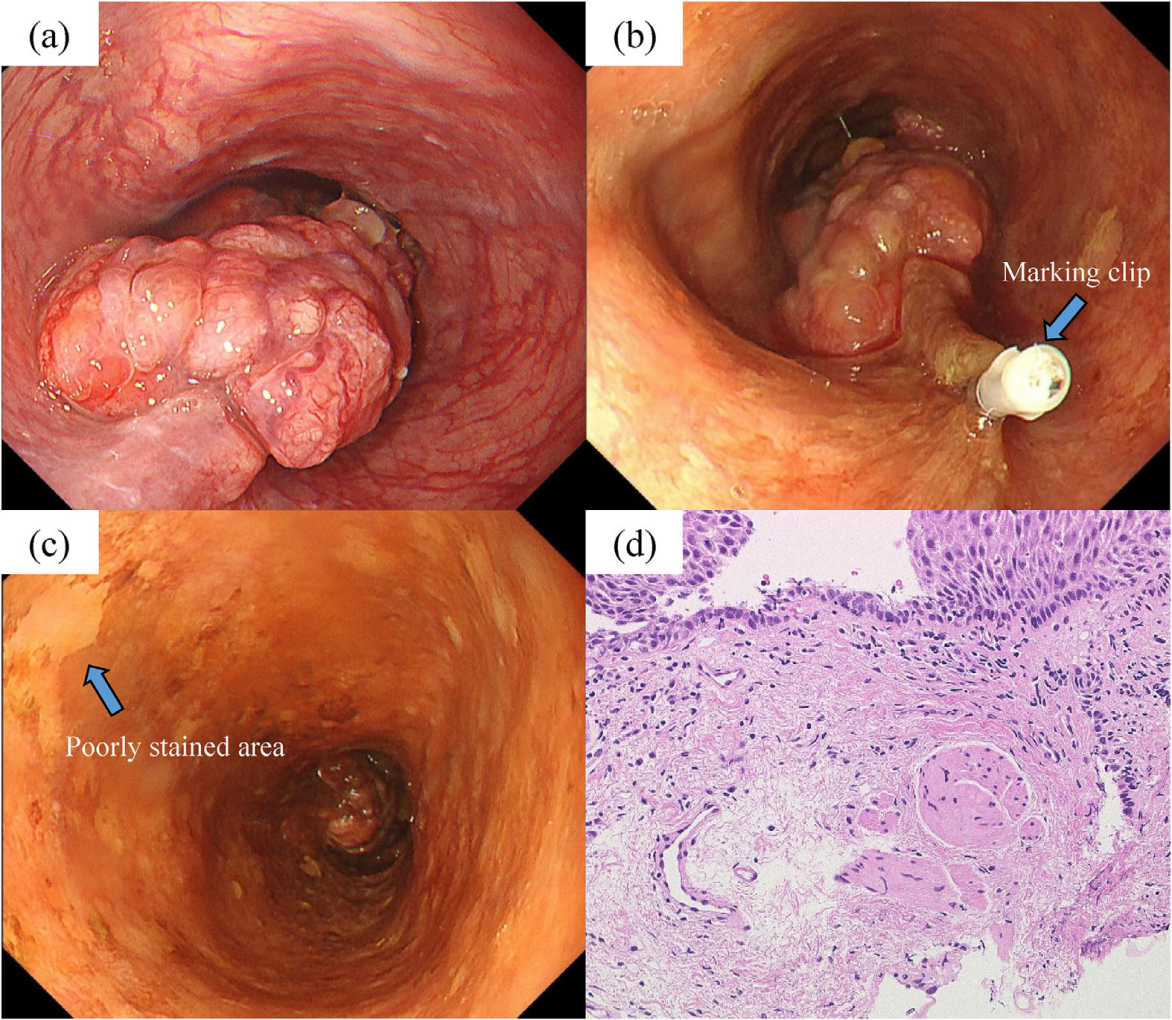
(a) The esophageal cancer extended from the upper thoracic to the mid‐thoracic esophagus, 26–32 cm from the incisors. (b) A marking clip was placed on the oral side of the lesion. (c) Iodine spraying revealed a poorly stained area 21 cm from the incisors, and a biopsy was performed. (d) Biopsy pathology revealed low‐grade squamous dysplasia and included muscularis mucosa.

A 70‐year‐old man was referred due to computed tomography (CT) findings of irregular esophageal thickening. He had experienced symptoms of esophageal obstruction for 2 months. His medical history included hypertension and postoperative left kidney cancer. However, no other conditions, such as pemphigoid, that could contribute to esophageal fragility were identified. Additionally, he was also not taking antithrombotic medications or undergoing long‐term steroid therapy. EGD revealed that esophageal cancer extended from the upper thoracic to the mid‐thoracic esophagus, 26–32 cm from the incisors (Figure 1a–c). The endoscope was able to pass through the area of the esophageal cancer without difficulty, and a marking clip was placed on the oral side of the lesion. Iodine spraying revealed a poorly stained area 21 cm from the incisors, and a biopsy was performed. Pathological findings showed that the lesion was low‐grade squamous dysplasia and the biopsy included muscularis mucosa (Figure 1d). No lymph node metastasis or distant metastasis was identified on contrast‐enhanced CT, leading to a diagnosis of cT3N0M0 stage II esophageal cancer. Surgical resection was selected as the treatment strategy, and barium esophagography was conducted immediately following the endoscopy in order to locate the esophageal cancer for preoperative evaluation. The esophagography showed temporary retention of barium in the upper thoracic esophagus due to compression by the tumor, and then flowed away. The patient vomited while ingesting barium for the third time.

**FIGURE 2:**
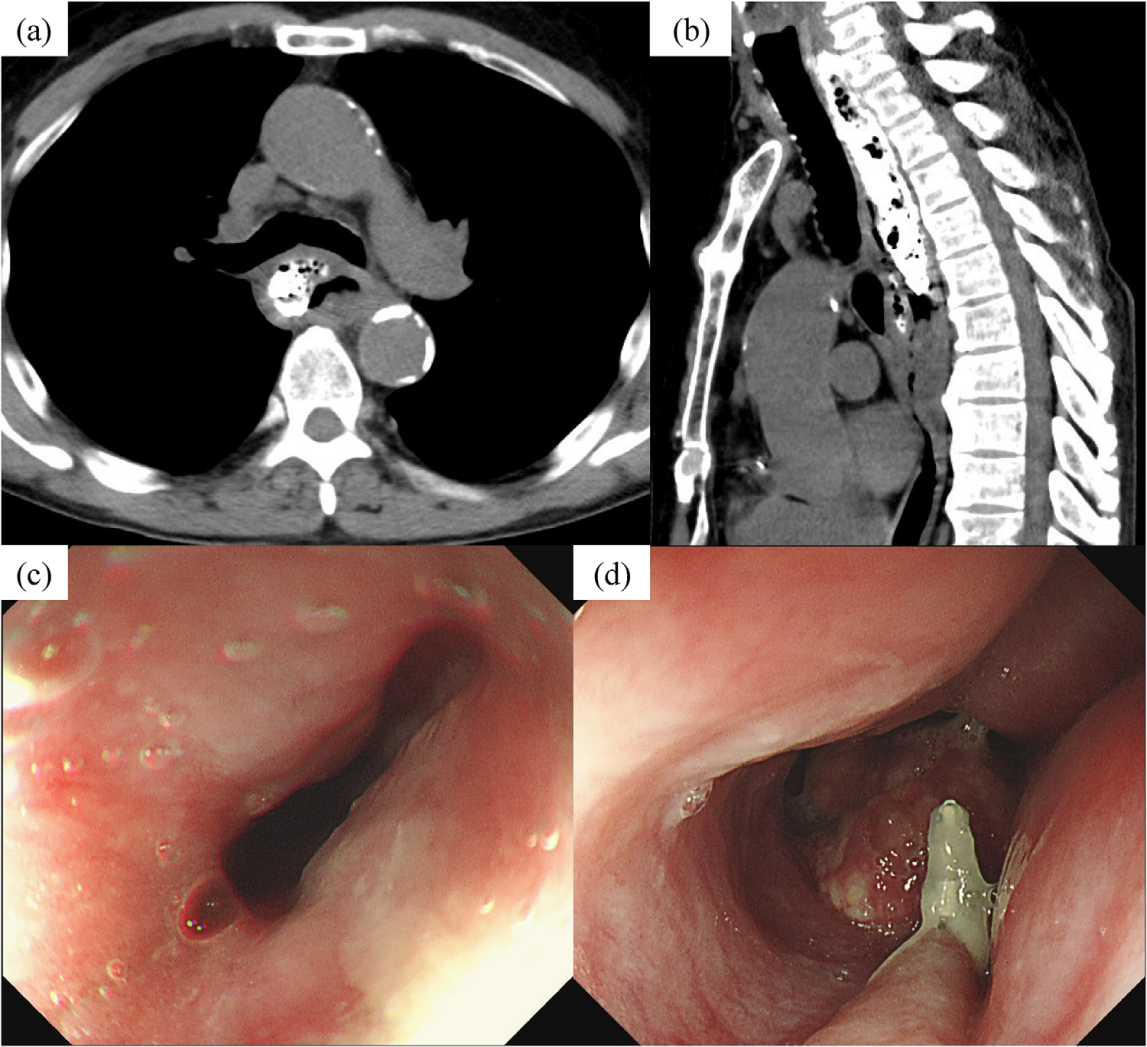
Day 3 after onset (a, b) A dissection cavity had formed surrounding the right and posterior walls from the cervical to the upper thoracic esophagus, filled with barium. No free air or barium leakage into the mediastinum was observed. (c, d) The cervical esophagus was elevated and the lumen was compressed. The mucosal surface of the elevated area was smooth, with no obvious hematoma.

After this episode, the patient experienced neck‐chest pain, as well as dysphagia, and sought medical attention three days later. CT revealed a dissection cavity filled with barium, surrounding the right and posterior esophageal walls from the cervical to the upper thoracic esophagus (Figure 2a,b). No free air or barium leakage into the mediastinum was observed, ruling out Boerhaave syndrome. EGD showed the cervical esophagus was elevated and the lumen was compressed (Figure 2c,d). The mucosal surface of the elevated area was smooth, with no obvious mucosal damage or hematoma, leading to a diagnosis of IED caused by vomiting during barium esophagography.

**FIGURE 3:**
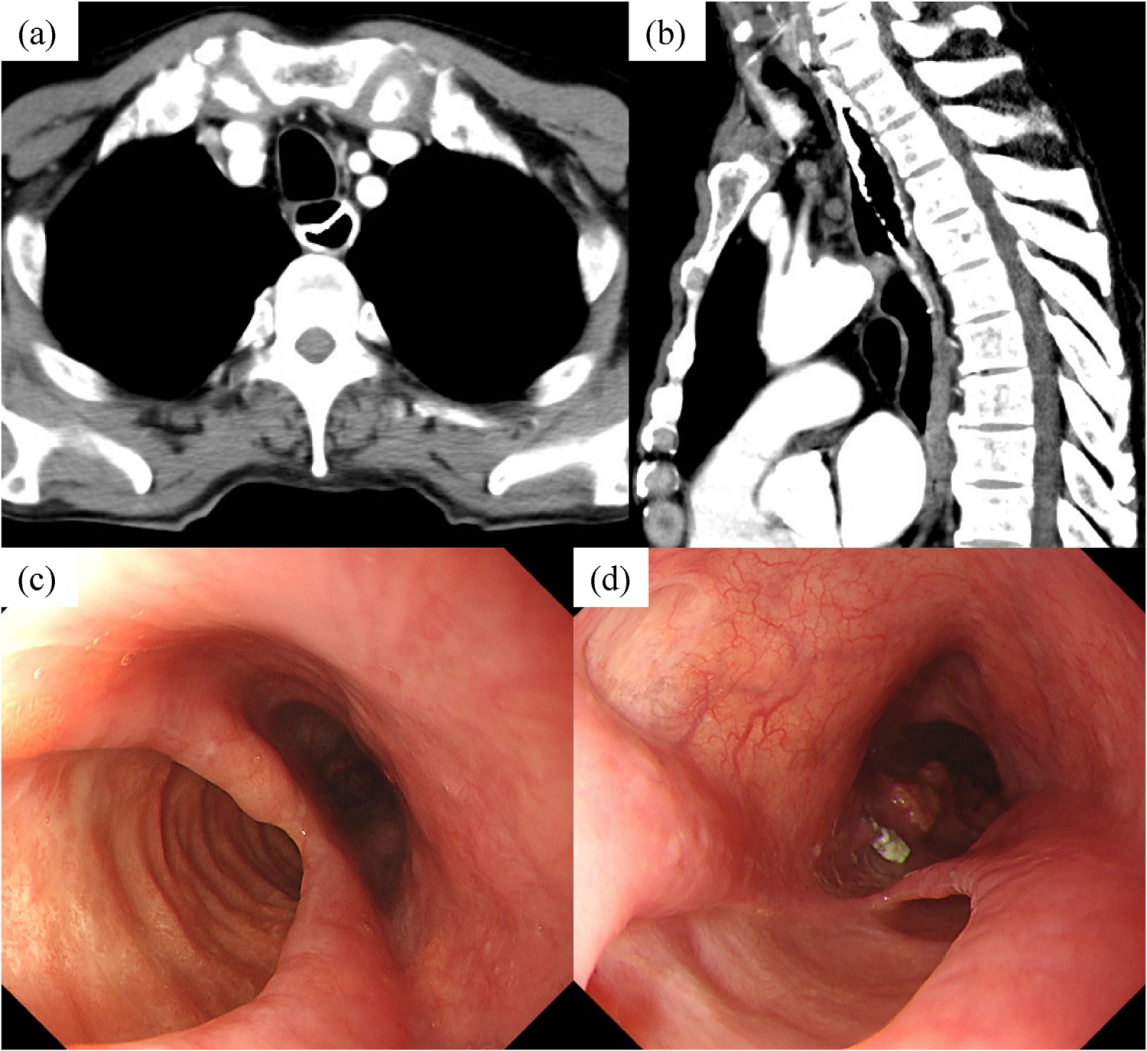
One month after onset (a, b) The dissection cavity was open and the barium had drained, forming a false lumen and a true lumen. (c, d) The entry site was 20 cm from the incisors, exposing the esophageal muscularis layer. The re‐entry site was located near the tumor, communicating with the dissection cavity.

The patient was treated conservatively with fasting and 2 g/day ceftriaxone. Symptoms improved without worsening, and inflammation markers decreased. One month later, a follow‐up CT and EGD were performed to assess his disease condition. CT revealed an open dissection cavity with drained barium, forming a false lumen and a true lumen (Figure 3a,b). EGD revealed that the entry site was 20 cm from the incisors, exposing the esophageal muscularis layer (Figure 3c,d). This was at the same level as the biopsy site of the poorly stained area. And the re‐entry site was located near the tumor, communicating with the dissection cavity.

**FIGURE 4:**
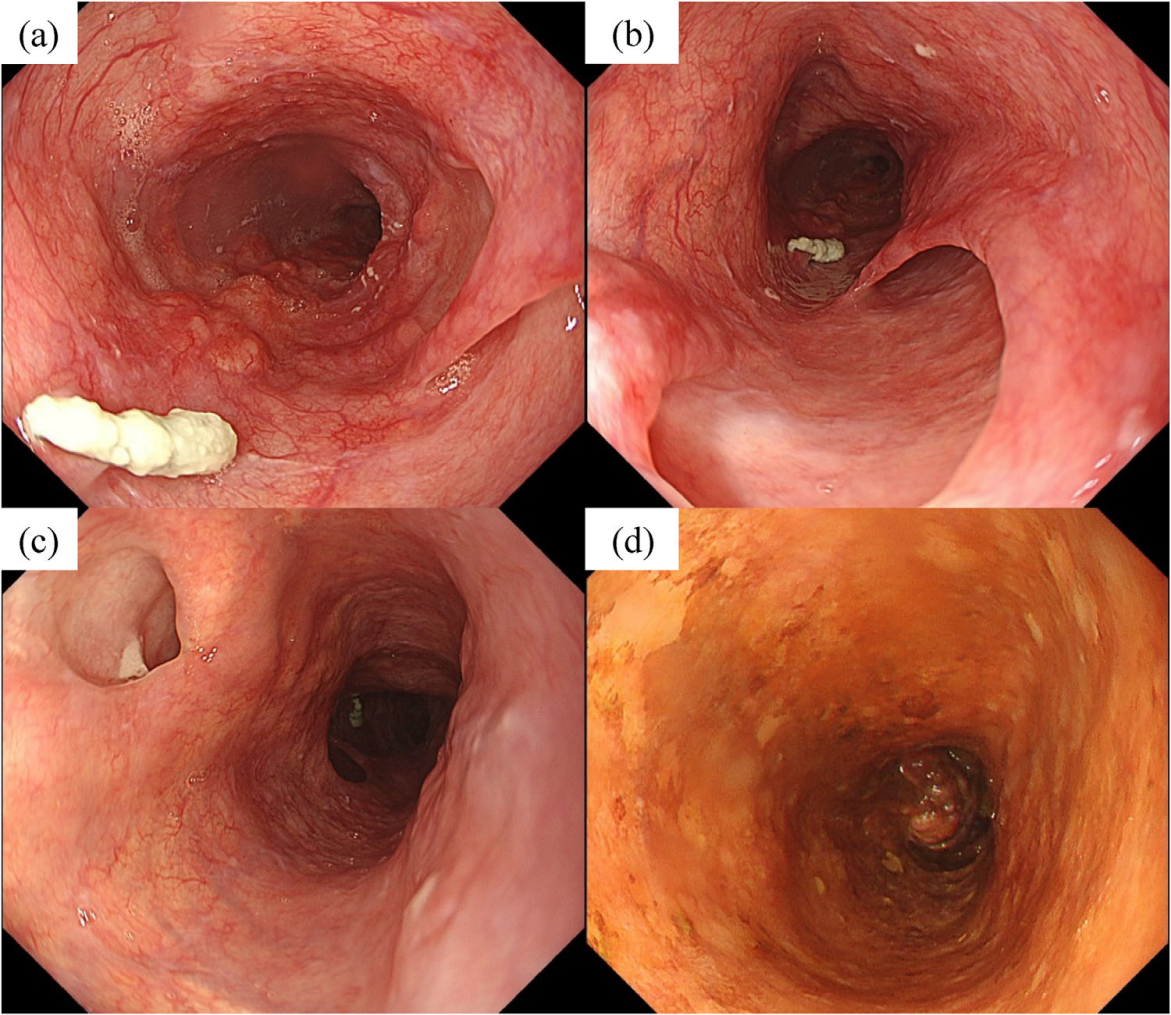
Four months after onset (a, b) The tumor volume of esophageal cancer decreased. The dissection lumen and the punched‐out ulcer were thin but epithelialized, forming a mucosal bridge. (c, d) The entry site of the dissection cavity was located at the same level as the biopsy site of the poorly stained area.

The patient no longer exhibited symptoms, such as dysphagia, and was able to eat without food residue accumulating in the dissection cavity, allowing the initiation of esophageal cancer treatment. Although surgical resection was considered, the patient's desire to preserve laryngeal function and the extension of the dissection to the cervical esophagus made this option challenging. Consequently, chemotherapy alone was chosen over chemoradiotherapy due to the risk of perforation from radiation. The patient received 5‐fluorouracil and cisplatin (FP). Two months later, EGD indicated a partial response (PR) with decreased tumor volume and no mucosal damage (Figure 4). The dissection lumen and ulcer were thin but epithelialized, forming a mucosal bridge, and the esophageal wound had healed completely. The patient remained on the FP regimen, with periodic EGD showing no worsening of IED and achieving long‐term PR for the esophageal cancer.

## DISCUSSION

IED was first reported by Williams^6^ in 1957. This condition is related to esophageal injuries like Mallory‐Weiss syndrome, which causes esophageal mucosal lacerations, and Boerhaave syndrome, which results in transmural esophageal perforation. Based on the cause of occurrence, it can be classified as spontaneous or traumatic. The spontaneous type is caused by self‐damage due to increased intraesophageal pressure induced by vomiting,^2^ while the traumatic type is caused by mechanical damage due to endoscopic intervention^3^ or accidental ingestion.^4^ In addition, patients with a history of antithrombotic drug use, hematologic disease, or hemorrhagic tendencies such as dialysis, are more likely to develop this condition.^5^

In the present case, neck‐chest pain developed after vomiting during the barium esophagography, suggesting that an increase in intraesophageal pressure was the trigger. The esophageal cancer was located in the upper thoracic esophagus at the level of the tracheal carina, which might have caused a stronger increase in internal pressure and vomiting reflex compared to a tumor in the lower thoracic or abdominal esophagus. In addition, before the barium esophagography, a marking clip was placed, and a biopsy for the poorly stained area was performed. Pathological findings revealed damage to the submucosal layer due to the biopsy scraping of the muscularis mucosae (Figure 1d), and the entry site of the dissection cavity was located at 20 cm from the incisors, at the same level as the biopsy site (Figure 4c,d). Therefore, it is speculated that the biopsy cut was torn by vomiting, leading to barium filling the dissection cavity due to increased internal pressure. Retrospectively, the poorly stained area approximately 5 mm in diameter without pink‐color signs is more likely to represent low‐grade squamous dysplasia. If we had been aware of the risk of IED in advance, the biopsy might not have been performed.

CT and EGD are considered useful for diagnosing IED,^7^, ^8^ and we were able to evaluated the wound process of IED over time from early onset. In the case of IED caused by esophageal submucosal hematoma, CT typically reveals a hyperabsorption zone within the dissection cavity suspected of hematoma, but in this case, the dissection cavity was filled with barium. EGD revealed that pressure from the dissection cavity caused elevation and compression of the esophageal mucosa immediately after the disease onset. Over time, the mucous membrane ruptured and the dissection cavity opened further, leaving some mucosa intact and forming a false lumen and a true lumen within the esophagus, resulting in a double‐barrel esophagus. A comprehensive search was conducted on PubMed using the keywords “intramural, esophageal, and dissection” which identified a total of 48 cases of IED diagnosed between 2004 and 2024. Among these, a false lumen remained in 20 cases following IED.

Conservative treatment with fasting is the principal therapy for this disease and may include mucosal protective agents or antacid agents such as proton pump inhibitors.^9^ The prognosis is relatively good, with esophageal ulcers covered by normal mucosa within 1–2 months and stenosis rarely observed. However, long dissection cavity where food residue tends to accumulate may become infected and abscessed. In such cases, the septum forming the dissection cavity can be cut off and left open.^10^ In the present case, however, since the wound healed without infection after the patient resumed eating, endoscopic treatment was not chosen. Furthermore, as the patient's condition was stable, chemotherapy was administered for esophageal cancer. Although there are no reports on treating esophageal cancer complicated by IED, we selected chemotherapy instead of chemoradiotherapy or surgery due to the risk of radiation perforation and preservation of laryngeal function. Chemotherapy did not cause mucosal injury and any other adverse events in the dissected cavity, achieving a PR.

In conclusion, we report a case of IED caused by vomiting during a barium esophagography following endoscopic biopsy in a patient with advanced esophageal cancer. This case suggests that, especially in patients with esophageal obstruction which can induce vomiting during esophagography, endoscopic biopsy could pose a risk of developing IED and should be carefully considered or potentially avoided.

## CONFLICT OF INTEREST STATEMENT

None.

## ETHICS STATEMENT

All procedures followed were performed in accordance with the ethical standards of the Declaration of Helsinki and its later amendments.

## PATIENT CONSENT STATEMENT

Informed consent was obtained from the patient for publication of this case report.

## CLINICAL TRIAL REGISTRATION

N/A.
